# Workshop report: Immunoassay standardisation for “universal” influenza vaccines

**DOI:** 10.1111/irv.12445

**Published:** 2017-04-08

**Authors:** Sophia Pavlova, Flavia D'Alessio, Sophie Houard, Edmond J. Remarque, Norbert Stockhofe, Othmar G. Engelhardt

**Affiliations:** ^1^ European Vaccine InitiativeUniversitätsKlinikum HeidelbergHeidelbergGermany; ^2^ Biomedical Primate Research CentreRijswijkThe Netherlands; ^3^ Wageningen Bioveterinary Research/Wageningen University & Re‐searchLelystadThe Netherlands; ^4^ National Institute for Biological Standards and ControlMedicines and Healthcare products Regulatory AgencySouth Mimms, Potters BarHertfordshireUK

**Keywords:** immunoassay, influenza, standardisation, universal, vaccine, workshop

## Abstract

The development of broadly reactive influenza vaccines raises the need to identify the most appropriate immunoassays that can be used for the evaluation of so‐called universal influenza vaccines and to explore a path towards the standardisation of such assays. More than fifty experts from the global influenza vaccine research and development field met to initiate such discussion at a workshop co‐organised by the EDUFLUVAC consortium, a European Union funded project coordinated by the European Vaccine Initiative, and the National Institutes of Health/National Institute of Allergy and Infectious Diseases, USA. The workshop audience agreed that it was not possible to establish a single immunoassay for “universal” influenza vaccines because the current approaches differ in the vaccines' nature and immunogenicity properties. Therefore, different scientific rationales for the immunoassay selection are required. To avoid dilution of efforts, the choice of the primary evaluation criteria (eg serological assays or T‐cell assays) should drive the effort of harmonisation. However, at an early phase of clinical development, more efforts on exploratory assessments should be undertaken to better define the immune profile in response to immunisation with new vaccines. The workshop concluded that each laboratory should aim towards validation of the appropriate immunoassays used during the entire process of vaccine development from antigen discovery up to establishment of correlates of protection, including the different steps of quality control (eg potency assays), animal studies and human clinical development. Standardisation of the immunoassays is the ultimate goal, and there is a long way to go.

## 1 Introduction

Current influenza vaccines afford only limited protection against antigenically drifted seasonal or against novel, pandemic influenza virus infection. Because of continuous antigenic changes of circulating influenza viruses, the seasonal influenza vaccine composition has to be updated regularly.

A significant advance in human infectious disease research would be the development of a new generation of influenza vaccines that induce a robust, broadly protective immune response, not only to drifted variants of seasonal influenza viruses, but preferably also to potential pandemic strains. Consequently, the development of such “universal” influenza vaccines has become a worldwide public health priority in both industrialised and low‐ and middle‐income countries.

A large number of academic, public or private organisations applying different approaches are developing such vaccines that are currently at various stages of development, from pre‐clinical evaluation to clinical trials.

The European Union funded EDUFLUVAC consortium, coordinated by the European Vaccine Initiative, co‐organised a workshop on the standardisation of immunoassays for “universal” influenza vaccines together with the National Institutes of Health/National Institute of Allergy and Infectious Diseases, USA. The main objectives of the workshop, held on 18‐19 June 2015, were (i) to review the immunoassays used to assess broadly reactive influenza vaccines; (ii) to explore a path towards standardisation of these assays; and (iii) to enhance networking and collaboration between the different actors interested in the development of novel influenza vaccines.

The workshop agenda, list of participants and presentations are published on the EDUFLUVAC website: http://www.edufluvac.eu/node/1117.

## 2 Immunoassays

### 2.1 Haemagglutination inhibition and single‐radial haemolysis assays

The immune response induced by influenza vaccines has been measured traditionally by three classical immunoassays: haemagglutination inhibition (HI), single‐radial haemolysis (SRH) and virus neutralisation (VN) assays. The HI assay detects antibodies that bind to the viral haemagglutinin and prevent the virus‐mediated agglutination of erythrocytes. There is a consensus, although not universally accepted, that an HI antibody titre of 40 correlates with a 50% reduction in the risk of contracting influenza^1^. This correlate of protection has been established from studies in healthy adults and is not appropriate for children^2^. The HI assay faces various technical challenges, such as variations when using erythrocytes from different species and variability between batches of erythrocytes from the same species. The antibody titres measured by HI and VN assays usually correlate somewhat for seasonal influenza viruses, but correlation may not be observed when testing non‐human subtypes such as H5N1 viruses.

The SRH assay measures antibodies that bind to the influenza virus and fix complement (usually guinea pig complement)^3^, ^4^. The SRH assay displays higher sensitivity for H5N1 viruses than the HI assay and has been shown to be relatively reproducible between laboratories. A correlate of protection has also been defined for SRH, that is a zone of 25 mm^2^ 5. While correlates of protection have been widely used for the HI and SRH assays, their relevance has been questioned and they are not valid in Europe any more^6^.

### 2.2 Virus neutralisation assay

Neutralising antibodies are generally accepted as primary mediators of immunity against influenza virus^7^. The VN assay mainly detects functional antibodies that bind the HA globular head and block receptor binding but can also measure antibodies that block entry at the stage of membrane fusion. There are various formats of the VN assay (Table 1).

**Table 1:** Formats of virus neutralisation assay

VN assay duration	VN assay read‐out
2 d	Enzyme‐linked immunosorbent assay (ELISA)
3‐7 d	Cytopathic effect
3‐7 d	Haemagglutination
3 d	Plaque reduction and focus reduction assays
3 d	Quantitative polymerase chain reaction (qPCR)

Collaborative studies have shown that interlaboratory variability of VN assays can be considerable; the use of antibody standards can reduce it^8^; however, antibody standards are not usually available for influenza serology studies.

As opposed to the HI assay, no correlate of protection has been established for the VN assay. In a recent household transmission study, microneutralisation titres ≥ 40 were associated with 49% protection against H3N2 infection^9^.

As the three main serologic assays HI, VN and SRH detect different populations of antibodies, different degrees of correlation between these assays can be observed.

### 2.3 Automated imaging of hemagglutination assays

Reading of haemagglutination and HI plates by operators is one cause of the variability of the HI assay. Different automated imaging analysis systems are currently under development and could standardise the reading of HI plates. The plate reader Cypher One^™^ was shown to achieve good correlation with expected titres in validation experiments. Moreover, there was good agreement between expert human readers and the automated reader. A high‐throughput machine is currently under development.

## 3 Analysis of Antibody Responses to the Influenza Virus Haemagglutinin Stalk Domain

Although antistalk antibodies can be produced after seasonal influenza vaccination, they comprise only a small proportion of total anti‐HA antibodies and are not efficiently boosted even after routine annual seasonal vaccination. However, anti‐HA stalk antibodies that have been isolated from humans (and mice) have been found to be cross‐reactive and neutralising between different subtypes and occasionally between group 1 and group 2 haemagglutinins^10^. There are a number of ways that antistalk antibodies may mediate immunity, including inhibiting viral fusion during entry, inhibiting new virion budding and mediating macrophage killing via Fc receptor (FcR) binding to antibody‐coated infected cells^11^. These different mechanisms inform the different types of serological assays that can be developed.

ELISAs can be used to detect total antistalk antibodies as well as heterosubtypic cross‐reactivity. The laboratory at Icahn School of Medicine at Mount Sinai has developed ELISAs using chimeric haemagglutinins^12^. These chimeras contain the stalk and globular head region from different influenza subtypes; thus, they can be used to measure stalk‐specific antibodies, eliminating interference from antiglobular head antibodies. Stalk epitopes require the HA1 subunit to be attached to the HA2 subunit in order to maintain epitope conformation. A number of HA stalk epitopes exist, and only two main epitopes have been characterised until now^13^.

Viruses generated via reverse genetics, containing chimeric haemagglutinins with mismatched neuraminidase genes, can also be used in anti‐HA‐stalk‐specific neutralisation assays. Plaque reduction neutralisation assays are the preferred format currently. Other microneutralisation assays are carried out in 96‐well format using nucleoprotein (NP) staining, haemagglutination assay and cytopathic effect end point read‐outs.

### 3.1 ELISA for influenza serology

An advantage of the ELISA is that a wide range of antigen preparations can be used as target antigens. Such preparations can be whole‐inactivated virus, subunit vaccines or purified HA1; therefore, antibodies towards specific regions or formulations of interest can be detected. In addition, IgG subclasses and immunoglobulin isotypes can be quantified in serum by the use of subclass/isotype‐specific conjugated secondary antibodies. ELISAs allow measuring the actual amounts of immunoglobulin, and the use of standards allows good intra‐ and interlaboratory standardisation. The ELISA method is also suitable for high throughput and is cost effective.

Comparison between HI assays and ELISAs has shown some correlation; however, the mean fold increase in HI titres is usually higher than in ELISA IgG titres. It should be noted that antibodies detected in HI assays comprise only a minor fraction of the total IgG antibody response. ELISA data were also compared to VN assay results in a clinical study of a non‐adjuvanted H5N1 vaccine candidate, showing correlation between HA‐specific IgG levels and VN titres^14^.

## 4 Approaches for the Evaluation of Antibody‐mediated Enhanced Infection

Some reports indicate that vaccine‐induced immune mediators may enhance disease upon subsequent influenza infection. Epidemiological observations have suggested that pre‐existing anti‐H1 antibodies were associated with enhanced disease (ED) in some people following infection with pandemic A(H1N1) virus in 2009^15^. Experimental studies in pigs supported these observations; piglets vaccinated and then challenged with heterologous H1 virus demonstrated ED which was not caused by neutralising antibodies^16^. The underlying mechanism appears to be mediated by anti‐HA2 cross‐reactive antibodies which are able to enhance viral fusion when present in the absence of vaccine‐induced anti‐HA1 neutralising antibodies^17^. Other proposed mechanisms for vaccine‐associated ED include low avidity antibodies forming immune complexes (complement binding)^17^, T cells and NK cells.

These results have relevance for assessing “universal” vaccines that have been designed to target the HA2 region. Studies should include screening for possible vaccine‐induced disease enhancement. Relevant in vitro assays include those measuring Fc‐mediated influenza virus uptake using relevant cell lines (eg human epithelial cells, monocytes). Proposed read‐outs are plaque assays or anti‐NP immunostaining, or real‐time PCR for viral RNA quantification in high‐throughput assays. In vivo studies in pigs can also be used to assess vaccine‐induced disease enhancement.

### 4.1 Influenza antibody profiling by protein microarray

An alternative approach to current serological assays is the use of protein microarrays that can simultaneously measure antibody responses to a variety of virus strains and subtypes while requiring only small amounts of test serum. The protein array method involves HA1 proteins of different influenza subtypes spotted in triplicate onto nitrocellulose pads on glass slides. The serum samples are then added in twofold serial dilutions, and a fluorescently conjugated detection antibody is used to visualise positive signals^15^. Variations between slides are controlled by using a serum standard, for example the international standard for antibody to influenza A(H1N1)pdm09 (NIBSC).

The protein array was successfully applied using pre‐ and post‐H1N1pdm09 human sera^15^. There was good agreement with HI assay in terms of positive responders. However, the two assays varied when serum was assayed over a time course whereby the protein array assay appeared to detect positive responses earlier. This may be because the protein assay can detect broader antibody responses which are present following multiple previous exposures. Interestingly, antibodies against H9 were also detected in some sera in this study even though it is unlikely these subjects would have been naturally exposed^15^. This is perhaps due to the increasing breadth of antibodies made against conserved epitopes over a person's lifetime that can cross‐react with multiple subtypes. Whether these antibodies offer any protection remains to be determined.

Future work should include more comparative experiments between the protein array and biological assays to assess the functional relevance of the protein array data.

## 5 Measurement of Antineuraminidase Inhibition Titres by Enzyme‐linked Lectin Assay

Traditionally, anti‐NA assays have used the thiobarbituric acid (TBA) method. To reduce the use of highly hazardous chemicals, a mini‐TBA assay has been established^18^. A more practical alternative assay for measuring human anti‐NA antibody titres is the enzyme‐linked lectin assay (ELLA)^19^.

The ELLA method consists of coating plates with fetuin, a substrate for NA, and adding test serum and virus and/or NA. Active NA cleaves the terminal sialic acid residue from the fetuin, leaving an exposed galactose molecule. Peanut agglutinin (PNA) conjugated to horseradish peroxidase is then added whereupon the PNA binds exposed galactose molecules. Results can be determined as 50% end point titres. ELLA and mini‐TBA assay results are comparable.

Variables within the ELLA assay include the source of the NA, for example purified NA, detergent disrupted virus or whole virus containing a mismatched HA. It may be necessary to heat treat the serum in order to reduce non‐specific inhibition. The amount of virus/NA added per well needs to be predetermined by titration. Ideally, this part of the assay needs to be standardised between laboratories.

The Consortium for the Standardisation of Influenza Seroepidemiology (CONSISE) is carrying out work on standardisation of the ELLA assay. The aim is to identify key variables that lead to interlaboratory and interassay variability and to evaluate a serum standard. Results to date showed that the antigen dilution appears to be critical; lower titre results were generated if too much antigen was added. Use of a standard serum improved comparability, at least for anti‐N1 specific responses. Updating the assay buffer to stipulate a pH that optimises NA activity can further improve the method.

Alternative anti‐NA serological assays, in addition to the established TBA and ELLA methods, are presented in Table 2.

**Table 2:** Neuraminidase assays

Assay	Characteristic	Pro	Contra
MU‐NANA 2′‐(4‐methylumbelliferyl)‐α‐D‐N‐acetylneuraminic acid) assay	MU‐NANA substrate is cleaved by NA, releasing a fluorescent 4‐methylumbelliferone molecule allowing measuring of anti‐NA antibody levels by inhibition of this cleavage and reduction in fluorescence	Quick and easy to perform	Highly variable; as MU‐NANA is not the natural substrate, the biological relevance is questionable
AVINA (Accelerated virus inhibition with NA read‐out) assay^19^	It detects anti‐HA and anti‐NA antibodies. It involves infecting permissible cell monolayers in the presence of test sera followed by testing the supernatants for NA activity using the MU‐NANA assay		
Western blot	Quantify anti‐NA antibodies binding NA antigen that can be derived from a number of sources: insect cell expression systems or split virus vaccines	Measures direct interactions between antigen and antibody	Issues with regard to maintenance of NA conformation
Plaque reduction assay	Essentially a plaque assay in the presence of test serum. Anti‐NA antibodies inhibit the growth of plaques on a permissive cell monolayer, for example MDCKs, overlaid with agar	Functional anti‐NA antibodies can be quantified; it may be best used to provide supplementary data	Very labour‐intensive and has long incubation times; potential interference of anti‐HA antibodies
Microneutralisation assay adapted for anti‐NA responses	MNT with viruses containing mismatched HA; cell lines that do not require addition of exogenous trypsin (eg CaCo 2 cells) are desirable as the addition of trypsin may affect sensitivity of assay readouts		
ELISA	Expressed or purified NA is used in an ELISA format	Easy to perform; measures direct interaction between antigen and antibody	No information about inhibitory activity of antibodies

## 6 Role of T and B cells in Response to Influenza Vaccines

Both T and B cells play a role in the generation of protective immune responses to influenza vaccines. CD4 T cells are needed for CD8 T‐cell memory as well as high‐affinity, antigen‐specific B‐cell development. Although influenza‐specific CD4 and CD8 T cells are generated against conserved influenza antigens/peptides, they do not appear to be generated at a level that is enough to afford protection year after year. However, pre‐existing memory CD4 T cells can enhance the early antibody response.^20^ Vaccination strategies could therefore include the use of conserved peptides to stimulate the formation of memory CD4 cells.

Human studies have shown a link between CD4 specificity and help for the neutralising antibody response. For example, people immunised with split H5N1 vaccines followed by immunisation with a homosubtypic heterologous H5N1 vaccine generated increased neutralising antibody titres as compared to people that were not primed, the majority showing levels above the predicted protective threshold of 1:40^21^. Thus, pre‐existing cross‐reactive CD4 T cells can be a limiting factor in the generation of protective antibody titres to influenza proteins. Peptides used to prime this CD4 response must be physically linked to those necessary for the corresponding B‐cell response. In terms of correlates of protection, the numbers of HA‐specific CD4 T cells present appear to correlate well with the neutralising antibody response to split virus and subunit vaccines.

It is likely that only a subset of the available memory CD4 T cells will be stimulated to make an effective response during vaccination. It should also be noted that some subsets may antagonise the response.

Peptides are available from Biodefense and Emerging Infections (BEI) Research Resources Repository/NIH that can be used in in vitro cellular assays. The need to focus on immunodominant peptides is expected as there are other regions of the influenza proteome that do not appear to stimulate adequate T‐cell responses.

Follicular helper T cells have been found in the blood which may be a correlate of influenza antibody responses^22^.

### 6.1 ELISpot

The enzyme‐linked immunosorbent spot (ELISpot) assay is used to quantify the numbers of antigen‐specific cytokine secreting cells after antigen stimulation. Typically, IFNγ is used as a measure of activated T cells. However, this assay is flexible and any immune cell types (T cells and B cells) and cytokines can be measured if appropriate detection antibodies are available. The procedure involves incubating antigen‐stimulated lymphocytes in 96‐well plates coated with cytokine‐specific antibody. Secreted cytokines are then detected using a biotinylated antibody, alkaline phosphatase‐conjugated streptavidin and a colour‐forming substrate to enable the visualisation of coloured spots on the plate.

ELISpot is a suitable assay for measuring T‐cell responses to influenza and has been used in a number of published studies that have investigated the T‐cell response during natural infection as well as after vaccination. Interestingly, studies have shown that H5N1‐reactive memory T cells are present in populations where H5N1 is not prevalent^23^. Instead, they appear to have been established by seasonal influenza infections. The majority of the cross‐reactive T cells were responsive against either the NP or M protein. ELISpot has been found to be a highly sensitive assay for the evaluation of memory T cells in children vaccinated with live attenuated vaccines; cell‐mediated immune responses measured by IFNγ ELISpot correlated with protection^24^.

Although the ELISpot assay performance is straightforward, there are a number of variables that must be considered for assay standardisation both within and between laboratories, for example peripheral blood mononuclear cell (PBMC) preparation procedures, reagents, incubation periods and the stimulating antigen used. It is also necessary to use clinical trial data and human subjects in order to ascertain correlates of protection.

### 6.2 Intracellular cytokine staining assay

The intracellular cytokine staining (ICS) assay can be used to evaluate T‐cell immunity and correlates of protection for influenza vaccines. This assay is currently being standardised through the European Union funded UNISEC project.

The ICS assay measures multiple cytokines and lymphocyte subsets and is designed to maximise the amount of data from the limited cell numbers available during clinical trials. This collection of broad data aims to ensure that the currently unknown relevant mechanisms of cell‐mediated immunity and associated correlates of immunity are likely to be captured. The assays for influenza are adapted from those established for the evaluation of HIV vaccines in clinical trials^25^. In brief, the procedure entails the collection and freezing of PBMCs using a standardised method, followed by thawing and stimulation using vaccine‐relevant antigens. Cells are stained for the surface markers CD3, CD4 and CD8 and then fixed and permeabilised to enable intracellular cytokine staining (eg IL‐2, IL‐4, IFNγ, TNFα). Positive cell subsets are measured using flow cytometry.

In the ICS assay, common peptides between subtypes could be used to measure potential universality. Currently, most available peptide pools for influenza are optimised for CD8, rather than CD4 cells.

With regard to standardisation, there is an element of subjectivity introduced by the operator when analysing flow cytometry data, for example the positioning of the gates around the cell populations of interest. This may warrant an automated analysis process.

Seeing as the influenza virus infects the lungs and that a non‐highly pathogenic influenza virus does not spread into the blood, it was suggested that it may be valuable to measure early responding T cells in the lungs rather than or in addition to PBMCs. Measuring neutrophils and natural killer (NK) cells may also be of interest. The kinetics of cytokine production should be considered when establishing this type of assay as cytokine production varies over time. Thus, time points post‐vaccination/infection need to be standardised and verified. It should be noted that the ELISpot assay can detect cytokine producing cells from samples taken over a long time because the cytokines accumulate in the cell.

### 6.3 Cytotoxicity assays

“Universal” and novel influenza vaccines frequently aim to target the cytotoxic T‐cell (CTL) response, and therefore, relevant assays are needed. It has been shown that CTL responses can be a valid correlate of protection against symptomatic influenza disease^26^.

CTLs recognise infected cells through T‐cell receptor interaction with major histocompatibility complex (MHCI) molecules coated with peptides, followed by target cell killing (by lysis and/or apoptosis mediated by release of perforin, granzyme and FasL expression). Activated CTLs also produce cytokines such as IFNγ and TNFα. A summary of established CTL assays is presented in Table 3.

**Table 3:** Cytotoxicity assays

Type of CTL assay	Characteristic	Pro	Contra
Classical proliferation assays using: 1 ‐ tritiated (3H) thymidine incorporation 2 ‐ 5,6‐Carboxy Fluorescein diacetate Succinimidyl Ester (CFSE) fluorescent dye labelling	CFSE is more common; no radioactive isotopes	Easy to handle	No functional data; bystander activation can occur
Chromium release assay	Measures lysis of target cells labelled with ^51^Cr, which is released upon antigen‐activated‐CTL killing	Functional data	Involves radioactive isotopes
Flow cytometry detection of degranulation	Measures degranulation cell surface marker CD107a; this can be coupled with IFNγ ICS detection following peptide pool stimulation of PBMCs	Functional data; safe, no radioactivity	
Fluorescent antigen‐transfected target (FATT)‐cells assay	GFP fusion of the gene of interest transfected into cells, followed by presentation of peptides of interest on MHCI; Co‐culturing of target cells with HLA‐matched CTLs and detection of subsequent killing by fluorescent nucleic acid stain TOPRO‐3 as a marker of cell viability	Functional data; safe, no radioactivity	Measurement of transfected rather than virally infected cells
Viral clearance assay	Involves infecting autologous lymphocytes with a recombinant virus expressing a reporter gene and co‐culturing them with serial dilutions of effector T cells; fluorescence is measured by fluorescence microscopy or flow cytometry		Use of live virus; challenging standardisation

### 6.4 Antibody‐dependent cellular cytotoxicity assay

The antibody‐dependent cellular cytotoxicity (ADCC) assay is a relatively new method to determine the combined antiviral effect of antibodies and Fc receptor‐bearing effector cells (NK, macrophages and neutrophils). The breadth of the antibody response detected was extended beyond classical neutralising (HI and VN) antibodies to antibodies recognising all viral surface proteins (HA, NA, M2) by the ADCC assay. Different ADCC methods^27^ are summarised in Table 4.

**Table 4:** ADCC methods

Method	Set‐up	Read‐out
Plate‐bound ADCC NK cell activation assay	Whole virus or specific proteins are coated onto 96‐well plates, antibody and NK cells added	FACS analysis for intracellular IFN‐γ and surface CD107a on NK cells
Antibody‐dependent cell‐associated viral elimination assay with primary cells	Autologous PBMC are infected with influenza virus, followed by the addition of antibodies	1 ‐ Detection of infected cells (anti‐NP antibodies) 2 ‐ NK cell degranulation (CD107a) by FACS
Antibody‐dependent cell‐associated viral elimination assay with cell lines	A549 cells (human respiratory epithelial cell line) are infected with influenza virus, resuspended and labelled for HLA class I, followed by adding of PBMC and antibodies	1 ‐ Detection of infected cells (anti‐NP Ab) 2 ‐ NK cell degranulation (CD107a) by FACS
ADCC reporter assay	User‐specific target cells and effector cells containing a reporter gene indicating effector cell activation	Detection of reporter gene

Several potential assay improvements could be considered: use of target cells stably expressing antigens; replacement of NP detection by reporter gene (eg fluorescent antigen‐transfected target cells (FATT‐CTL), bioluminescent reporter gene); replacement of primary NK cells by an NK cell line; and use of methods to use BAL fluids in ADCC. The biological relevance of ADCC antibodies and the potential use of ADCC as a correlate of protection are currently under investigation.

### 6.5 Antibody‐dependent respiratory burst assay

Antibody‐dependent respiratory burst (ADRB) assay is an assay to measure the combined (antiviral) effects of antibodies and neutrophils^28^. The assay was first described for malaria and was well correlated with protection^28^. The ADRB assay is a relatively simple technique to assess antibody functionality (similar to ADCC) requiring only luminescence equipment. The ADRB assay can be performed in high throughput, and it can be standardised. Two potential assay set‐ups^29^ are described in Table 5.

**Table 5:** ADRB assays

Method	Set‐up	Read‐out
Plate‐bound ADRB,	Whole‐virus (or specific) proteins are coated onto 96‐well plates, antibody and neutrophils added, followed by addition of isoluminol	Luminescence measurement
Infected cell ADRB	Target cells are infected with influenza virus, followed by the addition of antibodies, neutrophils and isoluminol	Luminescence measurement

The relevance of the ADRB for influenza is still being explored, and a number of issues remain to be investigated; these include the following: correlations between formats using plate‐bound antigen and those using infected cells and the use of a reference standard on every plate and correlation with other (ADCC, ELISA IgG) assays.

## 7 Discussion and Conclusion

In summary, establishing “universal” immunoassays for “universal” influenza vaccine(s) is challenging if the vaccines differ significantly in their nature and immunogenicity properties. Therefore, different scientific rationales regarding the decision of the immunoassays are required for the development of different “universal” influenza vaccines. To avoid dilution of efforts, the choice of the primary evaluation criteria (eg HI, VN, ELLA, ELISpot) should drive the effort for harmonisation. However, at an early phase of clinical development, more efforts on exploratory assessments should be undertaken to better define the immune profile.

Steps towards standardisation of immunoassays between laboratories are the harmonisation of assay protocols, use of common standards and regular proficiency assessment of laboratories. Although the harmonisation of assay procedures might be feasible, standardisation of critical reagents will be more difficult, if not impossible, primarily due to local sourcing of reagents. Harmonisation of methods does not always lead to improved agreement between laboratories, as demonstrated for the HI assay^30^.

Each laboratory should aim to appropriately validate the immunoassays used during the entire process of vaccine development from antigen discovery up to establishment of correlates of protection, including the different steps of quality control (eg potency assays), animal studies and clinical development. Global standardisation of the immunoassays is the ultimate goal; however, there is still a long way to go.

The CONSISE consortium, where more than 100 members from 40 countries share study and laboratory assay protocols and other information on the Internet with free access, is a good example of efforts towards the standardisation of immunoassays. CONSISE developed new consensus protocols for the HI and VN assays, and member laboratories have aligned their assay parameters to the consensus protocols.

There are different levels/types of standards:

1 ‐  WHO International Standards—highest level
2 ‐  National/regional/pharmacopoeial standards
3 ‐  Working reagents
4 ‐  In‐house standards
5 ‐  Run controls

Possible approaches towards harmonisation of immunoassays could be the establishment of antibody/serum standards in case the vaccine candidate targets antibody responses. However, they might need to be product‐specific if particular epitopes are targeted. Standards for specific biological analytes (eg cytokines) can also be used. Cell standards for cell‐mediated immunity assays have been made in the past and more could be generated.

Proficiency testing and external quality assessment are important for the evaluation of the process of harmonisation and standardisation. Testing a laboratory's quality system with samples of known, but undisclosed content will give an overview of the laboratory's performance and will provide a comparison with that of other laboratories.

A clear need for further collaboration, networking and international communication regarding the harmonisation of immuno‐assays was identified, and CONSISE could be the venue for these activities. The standardisation should be initiated early, before methods drift too much. The European‐American partnership established during the organisation of this workshop was recognised as a clear asset in this direction and will be sustained.
